# The Alzheimer's β-secretase enzyme BACE1 is required for accurate axon guidance of olfactory sensory neurons and normal glomerulus formation in the olfactory bulb

**DOI:** 10.1186/1750-1326-6-88

**Published:** 2011-12-28

**Authors:** Tharinda W Rajapaksha, William A Eimer, Thomas C Bozza, Robert Vassar

**Affiliations:** 1 Department of Cell and Molecular Biology, Feinberg School of Medicine, Northwestern University, Chicago, IL 60611, USA; 2 Department of Neurobiology, Northwestern University, Evanston, IL 60208, USA

**Keywords:** Aβ, BACE1, β-amyloid, β-secretase, Alzheimer's disease, axon guidance, axon targeting, glomerulus, odorant receptor, olfactory sensory neuron, olfactory bulb, olfactory system

## Abstract

**Background:**

The β-secretase, β-site amyloid precursor protein cleaving enzyme 1 (BACE1), is a prime therapeutic target for lowering cerebral β-amyloid (Aβ) levels in Alzheimer's disease (AD). Clinical development of BACE1 inhibitors is being intensely pursued. However, little is known about the physiological functions of BACE1, and the possibility exists that BACE1 inhibition may cause mechanism-based side effects. Indeed, BACE1^-/- ^mice exhibit a complex neurological phenotype. Interestingly, BACE1 co-localizes with presynaptic neuronal markers, indicating a role in axons and/or terminals. Moreover, recent studies suggest axon guidance molecules are potential BACE1 substrates. Here, we used a genetic approach to investigate the function of BACE1 in axon guidance of olfactory sensory neurons (OSNs), a well-studied model of axon targeting *in vivo*.

**Results:**

We bred BACE1^-/- ^mice with gene-targeted mice in which GFP is expressed from the loci of two odorant-receptors (ORs), MOR23 and M72, and olfactory marker protein (OMP) to produce offspring that were heterozygous for MOR23-GFP, M72-GFP, or OMP-GFP and were either BACE1^+/+ ^or BACE1^-/-^. BACE1^-/- ^mice had olfactory bulbs (OBs) that were smaller and weighed less than OBs of BACE1^+/+ ^mice. In wild-type mice, BACE1 was present in OSN axon terminals in OB glomeruli. In whole-mount preparations and tissue sections, many OB glomeruli from OMP-GFP; BACE1^-/- ^mice were malformed compared to wild-type glomeruli. MOR23-GFP; BACE1^-/- ^mice had an irregular MOR23 glomerulus that was innervated by randomly oriented, poorly fasciculated OSN axons compared to BACE1^+/+ ^mice. Most importantly, M72-GFP; BACE1^-/- ^mice exhibited M72 OSN axons that were mis-targeted to ectopic glomeruli, indicating impaired axon guidance in BACE1^-/- ^mice.

**Conclusions:**

Our results demonstrate that BACE1 is required for the accurate targeting of OSN axons and the proper formation of glomeruli in the OB, suggesting a role for BACE1 in axon guidance. OSNs continually undergo regeneration and hence require ongoing axon guidance. Neurogenesis and the regeneration of neurons and axons occur in other adult populations of peripheral and central neurons that also require axon guidance throughout life. Therefore, BACE1 inhibitors under development for the treatment of AD may potentially cause axon targeting defects in these neuronal populations as well.

## Background

Proteolytic processing of the amyloid precursor protein (APP) by the β- and γ-secretases produces the Aβ peptide that forms amyloid plaques and plays a central role in AD pathogenesis. The β-secretase has been identified as the transmembrane aspartic protease BACE1 [1-5]. BACE1 cuts APP to produce a membrane-bound C-terminal fragment C99 that is further processed by γ-secretase to generate Aβ. BACE1 gene deletion in mice abrogates Aβ generation. Thus, BACE1 is considered a prime therapeutic target for AD.

Although BACE1^-/- ^mice are viable and fertile [6,7], they exhibit hippocampus-dependent memory deficits [8-10] and display abnormal EEGs and seizures [11,12]. Moreover, BACE1^-/- ^mice have hypomyelination [13,14], and additional BACE1^-/- ^phenotypes may exist. The molecular mechanisms that underlie the complex phenotypes of BACE1^-/- ^mice are not fully understood. Taken together, data suggest that therapeutic BACE1 inhibition may produce mechanism-based side-effects, thus underscoring the importance of fully understanding BACE1 physiological functions.

Deficient processing of specific BACE1 substrates appears to be the cause of certain BACE1^-/- ^phenotypes. For example, BACE1^-/- ^hypomyelination results from reduced BACE1 cleavage of type III neuregulin-1 [13,14] and deficient BACE1 processing of β2 sodium channel subunit [12,15,16] may cause seizures. A growing list of BACE1 substrates exists [17] and more will likely be discovered. A recent unbiased proteomics screen for novel BACE1 substrates suggested that members of different axon guidance molecule families were processed by BACE1 [18]. BACE1 levels are highest in neurons [1-3], especially in presynaptic terminals [19]. These results suggest that BACE1 has an important function in neurons, potentially in the axonal or terminal compartment. Intriguingly, the highest levels of BACE1 in the OB are in OSN axon terminals in glomeruli [20], suggesting a role for BACE1 there.

The axonal projections from OSNs to the OB form a topographic map through axon guidance processes that have been extensively studied in rodents and that are amenable to investigation using gene targeted mice [21]. The olfactory epithelium (OE) of mammals consists of ~10^7^-10^9 ^OSNs that each project a single axon to the OB, the first relay of the olfactory system. The mammalian genome encodes ~1000 different odorant receptor (OR) genes [22]. Each OSN appears to express only one OR gene [23,24]. Therefore, depolarization of a given OSN identifies the specific OR that has been activated.

In addition, OSNs expressing a given OR gene are distributed randomly within distinct expression zones of the OE [23,24]. This essentially random arrangement of OSNs creates a problem for the brain to determine which OSN has been stimulated in the OE for identifying the specific OR that has been activated. To solve this problem, axons of OSNs expressing a given OR gene converge onto ~2 glomeruli in spatially defined regions of the OB, thus establishing a glomerular map that represents OR activation and hence odor quality [25-27]. Each OB has ~1000 distinct populations of axons that coalesce into ~2000 glomeruli creating a formidable axon guidance problem both during development and in the adult, since OSNs continually regenerate and project new axons to glomeruli throughout life.

Here, we have taken advantage of the well-studied OSN axon targeting to the OB and the availability of OR gene targeted mice to investigate the role of BACE1 in axon guidance. To do so, we bred BACE1^-/- ^mice with gene-targeted mice in which GFP is expressed from the loci of two ORs, MOR23 and M72, and OMP. We observed that many BACE1^-/- ^glomeruli had abnormal morphologies as compared to BACE1^+/+ ^glomeruli. Most notably, M72-GFP; BACE1^-/- ^mice exhibited mis-targeting of M72 OSN axons to multiple ectopic glomeruli, in contrast to a single glomerulus per convergence site for BACE1^+/+ ^M72 OSN axons. Together, these results suggest that BACE1 participates in axon guidance, at least in OSNs, and they imply the possibility that therapeutic inhibition of BACE1 for AD may result in axon mis-targeting in humans.

## Results

### BACE1^-/- ^OB glomeruli have abnormal morphology

We noticed during routine dissections that the OBs of BACE1^-/- ^mice appeared smaller than those of BACE1^+/+ ^mice. To quantify this, we weighted OBs and brains from ~2-5 month-old BACE1^-/- ^and BACE1^+/+ ^mice and normalized OB weight to total brain weight for each genotype to exclude any effects of growth retardation [28]. We found that the normalized weights of BACE1^-/- ^OBs were 88.0 ± 2.74 (SEM)%, compared to 100 ± 3.17 (SEM)% for BACE1^+/+ ^OBs (p = 0.00856). Although this weight difference was small, it was statistically significant and it could not be attributed to decreased total brain weight for BACE1^-/- ^mice, and therefore it suggested that BACE1 deficiency had a selective effect on OBs.

Targeted gene deletions and manipulations (e.g., naris occlusion) that reduce peripheral olfactory input decrease OB size [29-33]. Therefore, the reduced size and weight of BACE1^-/- ^OBs strongly suggested that OSN axon guidance was perturbed, particularly since BACE1 is not abundant in the OB, except in OSN axon terminals [20]. Because axon guidance molecules were reported to be BACE1 substrates [18], we investigated whether the BACE1^-/- ^OB phenotype was related to impaired OSN axon formation or guidance. To initially test this, we bred BACE1^-/- ^mice with gene-targeted mice in which GFP is expressed from the locus of OMP (OMP-GFP mice) [34] to generate heterozygous OMP-GFP mice that were either BACE1^-/- ^or BACE1^+/+^. These mice displayed GFP labeling in all OSN axons and their terminals. Therefore, the entire olfactory nerve layer and all glomeruli of the OBs of these mice were labeled with GFP.

**Figure 1:**
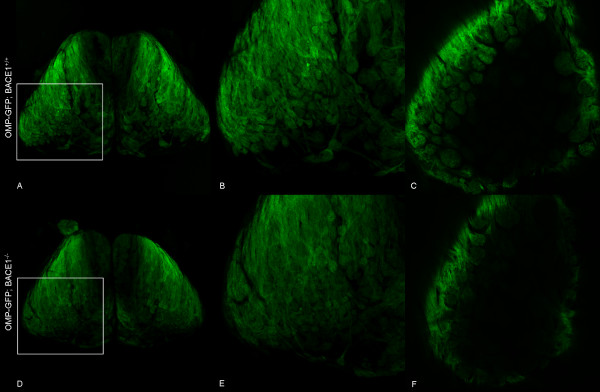
**Reduced clarity of OSN axon bundles and glomeruli in BACE1^-/- ^OBs**. Whole-mount preparations of OMP-GFP; BACE1^+/+ ^(A, B, C) and OMP-GFP; BACE1^-/- ^(D, E, F) OBs from 30 day-old mice were imaged for GFP fluorescence by laser scanning confocal microscopy. Z-stacks of optical sections were collected and collapsed into single images (A, B, D, E). Higher magnification images (B, E) of boxed regions in (A, D) show decreased clarity of OSN axons and reduced definition of glomeruli in the BACE1^-/- ^OB as compared to BACE1^+/+^. Abnormal morphologies of BACE1^-/- ^relative to BACE1^+/+ ^glomeruli are particularly evident in individual optical sections taken at a depth of ~110 μm from the dorsal surfaces of OBs (C, F). Note that the BACE1^-/- ^OB appears smaller in overall size relative to BACE1^+/+ ^(compare D to A). Images were taken of the dorsal OB surfaces and are shown with the anterior tips of the OBs at the top.

Whole-mount laser scanning confocal microscopy of OMP-GFP; BACE1^-/- ^and OMP-GFP; BACE1^+/+ ^OBs confirmed the smaller size of BACE1^-/- ^OBs (compare Figures 1A and 1D). When viewed at higher magnification, individual OMP-GFP; BACE1^-/- ^OSN axon bundles and glomeruli exhibited diminished visual clarity as compared to those of OMP-GFP; BACE1^+/+ ^OBs (compare Figures 1B and 1E), suggesting that the anatomical organization or structure of BACE1^-/- ^OSN axons and glomeruli was perturbed. Abnormal morphologies of OMP-GFP; BACE1^-/- ^glomeruli were particularly evident in individual confocal optical sections (compare Figures 1C and 1F), suggesting that BACE1 deficiency caused glomerular malformation.

**Figure 2:**
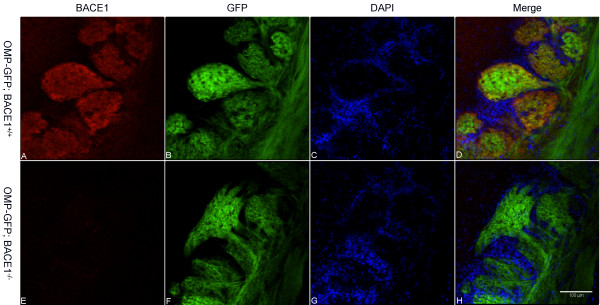
**Malformation of BACE1^-/- ^OB glomeruli**. Coronal sections of OBs from 60 day-old OMP-GFP; BACE1^+/+ ^(A-D) and OMP-GFP; BACE1^-/- ^(E-H) mice were immunostained with anti-BACE1 antibody and imaged for BACE1 (red; A, E), GFP (green; B, F), and DAPI for periglomerular cell nuclei (blue; C, G) by laser scanning confocal microscopy. Three-color image merges are shown in (D, H). Robust GFP fluorescence is observed in OSN axons and terminals in glomeruli (B, F). Note significant BACE1 co-localization with GFP in OSN terminals in BACE1^+/+ ^glomeruli (D). Importantly, abrogation of BACE1 expression results in markedly deformed BACE1^-/- ^glomeruli (F, H) as compared to BACE1^+/+ ^(B, D). Scale bar in (H) = 100 μm.

We next prepared coronal sections and performed laser scanning confocal microscopy of OMP-GFP; BACE1^-/- ^and OMP-GFP; BACE1^+/+ ^OBs to investigate the morphology of BACE1^-/- ^glomeruli in greater detail (Figure 2). GFP signal in OMP-GFP; BACE1^-/- ^OB sections revealed that many BACE1^-/- ^glomeruli were markedly deformed (Figure 2F), as compared to the typical spherical/elliptical shape of BACE1^+/+ ^glomeruli (Figure 2B). A significant amount of co-localization of the signals for GFP and anti-BACE1 antibody immunostaining demonstrated the presence of BACE1 in OSN axon terminals of BACE1^+/+ ^OB glomeruli (Figure 2A-D). BACE1 immunofluorescence signal was absent from OMP-GFP; BACE1^-/- ^OB sections, as expected (Figure 2E). Taken together, these data suggested that genetic abrogation of BACE1 expression in OSN axons resulted in a higher frequency of malformed OB glomeruli.

### OSNs expressing specific ORs exhibit axon guidance defects in BACE1^-/- ^OBs

**Figure 3:**
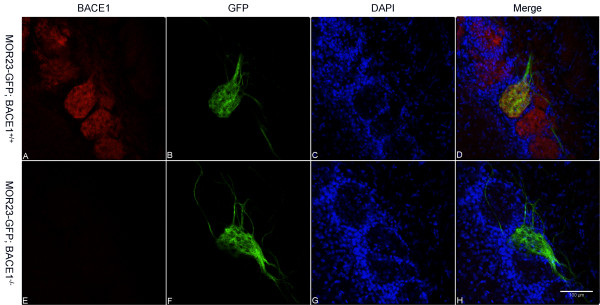
**Atypical OSN axon innervation of MOR23 glomerulus in BACE1^-/- ^OBs**. Coronal sections of OBs from 60 day-old MOR23-GFP; BACE1^+/+ ^(A-D) and MOR23-GFP; BACE1^-/- ^(E-H) mice were immunostained with anti-BACE1 antibody and imaged for BACE1 (red; A, E), GFP (green; B, F), and DAPI for periglomerular cell nuclei (blue; C, G) by laser scanning confocal microscopy. Three-color image merges are shown in (D, H). Robust GFP fluorescence is observed in OSN axons and terminals in MOR23 glomeruli (B, F). Note that largely individual BACE1^-/- ^OSN axons enter the MOR23 glomerulus from multiple directions and orientations (F, H), while most BACE1^+/+ ^OSN axons penetrate the MOR23 glomerulus as a bundle perpendicular to the glomerular layer (B, D), suggesting that BACE1 deficiency results in abnormal glomerular innervation. Scale bar in (H) = 100 μm.

One interpretation of our results with OMP-GFP; BACE1^-/- ^mice was that OSN axon guidance was impaired by BACE1 deficiency. To investigate whether BACE1 gene deletion perturbed the axonal projections of OSNs that express a specific OR, we employed gene-targeted mice in which GFP is expressed from the locus of the OR called MOR23 (MOR23-GFP mice) [35]. These mice display GFP labeling in MOR23 OR-expressing OSN axons, which project to only one topographically fixed glomerulus on each side of an individual OB. We bred BACE1^-/- ^mice with MOR23-GFP mice to produce heterozygous MOR23-GFP mice that were either BACE1^-/- ^or BACE1^+/+ ^and prepared coronal OB sections for immunofluorescence confocal microscopy (Figure 3). MOR23-GFP; BACE1^-/- ^OB sections revealed that the MOR23 glomerulus was irregular compared to wild-type. In contrast to the BACE1^+/+ ^MOR23 glomerulus in which OSN axons were organized mainly as a bundle that entered perpendicular to the glomerular layer, BACE1^-/- ^MOR23 OSN axons innervated the glomerulus mostly as individual axons at oblique angles from multiple directions (compare Figure 3F to 3B). As with OMP-GFP; BACE1^+/+ ^OB sections, GFP signal in BACE1^+/+ ^MOR23 axons co-localized well with anti-BACE1 immunostaining (Figure 3A-D). The randomly oriented, poorly fasciculated BACE1^-/- ^MOR23 OSN axons implied that BACE1 deficiency affected processes related to OSN axon guidance.

**Figure 4:**
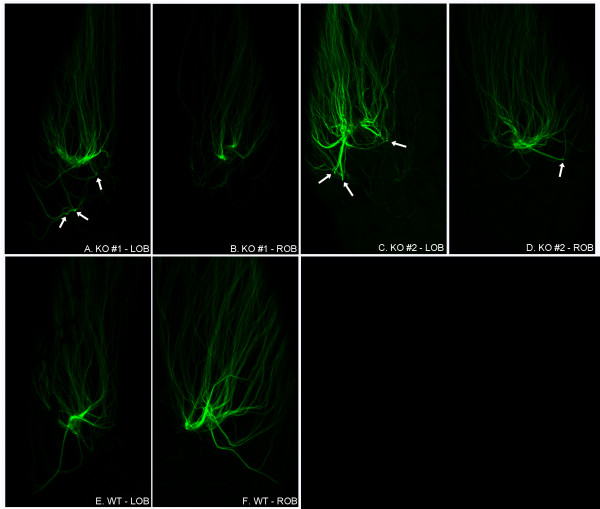
**M72 OSN axons terminate at ectopic sites in BACE1^-/- ^OBs**. Whole-mount preparations of M72-GFP; BACE1^-/- ^(A-D) and M72-GFP; BACE1^+/+ ^(E, F) OBs from 30 day-old mice were imaged for GFP fluorescence by laser scanning confocal microscopy. Z-stacks were collected and then collapsed into single images. Shown are 10× images of left (L) and right (R) OBs from two M72-GFP; BACE1^-/- ^mice (KO#1, KO#2) and one M72-GFP; BACE1^+/+ ^mouse (WT). M72 OSN axons from all BACE1^+/+ ^and BACE1^-/- ^mice innervate a major glomerulus. However, subsets of BACE1^-/- ^M72 OSN axons also target ectopic sites in the OBs that are near, but not within, the M72 glomerulus (arrows in A, C, and D). Although ectopic sites are not prominent in KO#1-ROB, the pattern of axon targeting to the M72 glomerulus appears abnormal, with two sites of innervation (B). In addition, OSN axons of KO#2-LOB appear to innervate two major glomeruli instead of one (C). Note that pigment cells obscure some regions of OSN axons in the WT-LOB image (E). Dorsal OB surfaces are shown with the anterior OB at the top of each image.

To determine whether BACE1 gene deletion could cause axon guidance defects in OSNs expressing a different OR, we used gene-targeted mice in which GFP is expressed from the locus of the OR called M72 (M72-GFP mice) [34]. We bred M72-GFP mice to BACE1^-/- ^mice to produce heterozygous M72-GFP mice that were either BACE1^-/- ^or BACE1^+/+ ^and then imaged their OBs by whole-mount confocal microscopy as before (Figure 4). The M72-GFP mice had OSN axons that targeted a major glomerulus, however all the BACE1^-/- ^mice also exhibited a subset of mis-targeted M72 OSN axons that innervated one or more ectopic sites in the OB that were near but not located within the M72 glomerulus (Figure 4A-D). As expected, all the M72-GFP; BACE1^+/+ ^mice displayed only the single M72 glomerulus with no ectopic sites innervated (Figure 4E, F). Thus, the OSN axon guidance defect caused by BACE1 deficiency was relatively more severe for M72 than MOR23.

**Figure 5:**
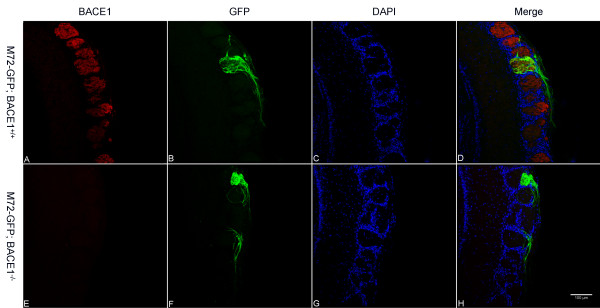
**M72 OSN axons are mis-targeted to ectopic glomeruli in BACE1^-/- ^OBs**. Coronal sections of OBs from 60 day-old M72-GFP; BACE1^+/+ ^(A-D) and M72-GFP; BACE1^-/- ^(E-H) mice were immunostained with anti-BACE1 antibody and imaged for BACE1 (red; A, E), GFP (green; B, F), and DAPI for periglomerular cell nuclei (blue; C, G) by laser scanning confocal microscopy. Three-color image merges are shown in (D, H). M72 OSN axons innervate a single glomerulus in the BACE1^+/+ ^OB (B, D). In contrast, BACE1^-/- ^M72 OSN axons target a major glomerulus (upper) and a minor glomerulus (lower) that are separated by ~3-4 intervening glomeruli (F, H). Note the presence of a single mis-targeted axon that wraps around the glomerulus adjacent to the major BACE1^-/- ^M72 glomerulus (F, H). These results strongly suggest that BACE1 deficiency causes axon guidance defects, at least for M72 expressing OSNs. Scale bar in (H) = 100 μm.

To investigate the M72 OSN axon targeting defect of BACE1^-/- ^mice in greater detail, we prepared coronal OB sections from M72-GFP; BACE1^-/- ^and M72-GFP; BACE1^+/+ ^mice and performed immunofluorescence confocal microscopy as above (Figure 5). As expected, M72 BACE1^+/+ ^OSN axons innervated a single M72 glomerulus (Figure 5B, D). Confirming our whole-mount results, we observed that most BACE1^-/- ^M72 OSN axons targeted a major glomerulus (Figure 5F, H). However, also consistent with the whole-mount data, a subset of BACE1^-/- ^M72 OSN axons displayed mis-targeting to ectopic glomeruli that were nearby but distinct from the main M72 glomerulus (Figure 5F, H). Although the OR specificities of the ectopic glomeruli have yet to be determined, these results demonstrate that BACE1 participates in OSN axon guidance and that BACE1 deficiency increases the error rate of OSN axon targeting, at least for M72 expressing OSNs.

## Discussion

Using a genetic approach, we determined the effects of BACE1 deficiency on OSN axon guidance using mice that have GFP expression coupled to the expression of a universally expressed OSN protein, OMP, and two different ORs, MOR23 and M72. We found that BACE1 plays a role in maintaining the accuracy of OSN axon targeting to specific OR glomeruli in the OB, and is also required for the normal morphological formation of OB glomeruli, at least for some OR specificities. BACE1 is present in OSN axon terminals in glomeruli, at the precise location where it can exert influence over axon guidance processes. In addition, BACE1^-/- ^OBs are smaller and weigh less than wild-type OBs. Taken together, these results strongly suggest that BACE1 has a function in certain aspects of axon guidance.

BACE1 has a large and growing list of known substrates [17], and it is likely that many others exist that have yet to be discovered. BACE1^-/- ^mice have a complex phenotype that is presumably derived from the summed effects of deficient processing of the entire complement of BACE1 substrates expressed throughout the body. Since neurons express the highest levels of BACE1 [1-3], we have focused on the neuronal substrates of BACE1. A recent unbiased proteomics screen uncovered a large number of novel putative BACE1 substrates [18], but most have yet to pass *in vivo *validation in BACE1^-/- ^mice. Intriguingly, a number of these putative BACE1 substrates are members of families of proteins that participate in axon guidance, including semaphorins, roundabout homologs, ephrins and Eph receptors, netrin receptor, NCAMs, protocadherins, and syndecans. An important first step in understanding the molecular basis of the role of BACE1 in axon guidance will be to determine which if any of these putative BACE1 substrates are processed by BACE1 *in vivo *and which are responsible for impaired OSN axon targeting in BACE1^-/- ^mice. In addition, it will be of interest to determine the functional consequences of BACE1 deficiency on olfactory perception.

## Conclusions

We conclude from our study that BACE1 has a role in axon guidance, at least in targeting OSN axons to glomeruli of the OB. It is currently unknown whether BACE1 participates in the guidance of axons in other neural systems of the body. Since several putative BACE1 substrates are widely expressed in both peripheral and central neurons, as is BACE1, one may speculate that BACE1 could influence axon guidance in multiple neural systems. In BACE1^-/- ^mice, compensation from other proteases may occur during development to rescue most axon targeting defects. Once stable connections are made, axon guidance, and hence a role for BACE1, may no longer be required. However, OSNs continually regenerate and thus require axon guidance, and BACE1, throughout life. Importantly, the axons of many adult PNS neurons can regenerate following injury and, together with adult CNS neural progenitor cell populations, also require ongoing axon guidance. Hence, the function of BACE1 in axon guidance may be required well into old age, precisely when human beings are at risk for AD. If BACE1 participates in the axon guidance of certain adult neuron populations, as our study suggests, then the therapeutic inhibition of BACE1 for AD may produce untoward side effects in those neurons. It remains to be seen whether this concern is justified, however our findings indicate that current and future clinical trials with BACE1 inhibitor drugs should incorporate tests for symptoms that might indicate dysfunction in neurogenic and/or axon regenerating neuronal systems.

## Methods

### Mice

BACE1^-/- ^mice [36] were purchased from Jackson Laboratory (Bar Harbor, ME) (stock number 004714). OMP-GFP [34], MOR23-GFP [35], and M72-GFP [34] mice have been reported previously. OMP-GFP is a gene-targeted mouse strain in which GFP is expressed from the locus encoding OMP. MOR23-GFP (originally referred to as MOR23-IRES-tauGFP) is a gene-targeted mouse strain harboring an IRES-tau-GFP fusion gene inserted 3' of the MOR23 OR gene coding region. M72-GFP (originally referred to as M72-IRES-tauGFP) is a gene-targeted mouse strain harboring an IRES-tau-enhanced green fluorescent protein (EGFP) fusion gene inserted 3' of the OR 160 (M72) gene coding region. BACE1^-/- ^mice were bred to each of the above strains to produce heterozygous OMP-GFP, MOR23-GFP, or M72-GFP mice that were either BACE1^+/+ ^or BACE1^-/-^. Breeding colonies of mice were maintained in the Northwestern University Center for Comparative Medicine animal facilities. All animal procedures were in strict accordance with the NIH Guide for the Care and Use of Laboratory Animals and were approved by the Northwestern University Animal Care and Use Committee.

### Antibodies and reagents

Anti-BACE1 rabbit monoclonal antibody (D10E5) was purchased from Cell Signaling (Boston, MA). Alexa Fluor 568-conjugated donkey anti-rabbit secondary antibody was purchased from Invitrogen (Carlsbad, CA). DAPI was purchased from Invitrogen (Carlsbad, CA).

### Whole-mount confocal microscopy

Mice were euthanized with carbon dioxide gas and the skull removed to expose the dorsal surfaces of the olfactory bulbs. The dissected head was stabilized dorsal side-up in a dish with Phosphate Buffered Saline. Unfixed whole-mount olfactory bulbs were imaged for GFP fluorescence on a laser scanning confocal microscope (Carl Zeiss LSM5 Pascal, Germany) with a 488 nm laser line and z-stack images were collected. Images were obtained within 30 minutes after euthanasia.

### Immunofluorescence confocal microscopy

Mice were deeply anesthetized with intraperitoneal injection of ketamine (200 mg/kg)/xylazine (25 mg/kg) and then transcardially perfused with 10% formalin in Phosphate Buffered Saline (PBS) before removal of olfactory bulbs. Both olfactory bulbs and the attached hemibrains were fixed in 10% formalin in PBS for 24 h and then cryopreserved in subsequent 24-hour cycles of 15%, 20% and 30% (w/v) sucrose, over a 72-hour period. 30 μm coronal sections were cut from cryopreserved olfactory bulbs on a freezing sliding microtome and collected in 0.1 M TBS. Free-floating OB sections were blocked in 5% donkey serum for 90 minutes on a rocking platform. Sections were incubated in 1:250 dilution of primary antibody against BACE1 (D10E5, Cell Signaling, Boston, MA) in TBS + 0.25% Triton-X 100 (TBS-T), 1% bovine serum albumin (BSA) in an incubator shaker at 37°C, 100 rpm for 2 hours. Sections were then washed in TBS-T with 1% BSA and incubated in 1:500 dilution of donkey anti-rabbit Alexa Fluor 568 secondary antibody and 300 nM DAPI in TBS-T with 1% BSA for 90 minutes at room temperature on a rocking platform. Sections were washed in TBS and mounted on slides. Coverslips were applied with Prolong Gold anti-fade mounting media (Invitrogen, Grand Island, NY). Images of the immunostained sections were acquired with a 20× air objective on either a Nikon C1Si or a Nikon A1 laser scanning confocal microscope using laser lines of 405 (blue) 488 (green) 561 (red) (Tokyo, Japan). Laser power percentage, gain, and offset settings were held constant for all images acquired and saturation was never reached. All images were acquired during one continuous session to prevent effects of decay of laser intensity.

## List of abbreviations

Aβ: β-amyloid; AD: Alzheimer's disease; APP: amyloid precursor protein; BACE1: β-site amyloid precursor protein cleaving enzyme 1; BSA: bovine serum albumin; DAPI: 4',6-diamidino-2-phenylindole; GFP: green fluorescent protein; NCAM: neural cell adhesion molecule; OB: olfactory bulb; OE: olfactory epithelium; OMP: olfactory marker protein; OR: odorant receptor; OSN: olfactory sensory neuron; PBS: phosphate buffered saline; SEM: standard error of the mean; TBS: tris buffered saline; TBS-T: tris buffered saline triton.

## Competing interests

The authors declare that they have no competing interests.

## Authors' contributions

TWR bred mice, performed whole-mount experiments, performed OB section experiments and confocal microscopy, assembled figures, and wrote the Methods section. WAE performed confocal microscopy and assisted in figure preparation. TCB provided OMP-GFP, MOR23-GFP, and M72-GFP mice, participated in the study design, trained and helped perform whole-mount experiments, and participated in the interpretation of results. RV conceived of the study, participated in its design and coordination, interpreted results, and drafted the manuscript. All authors read and approved the final manuscript.
